# BEYOND SPASTICITY REDUCTION: BOTULINUM TOXIN-A INJECTION IS ASSOCIATED WITH CHANGES IN ABNORMAL FLEXOR SYNERGIES OF THE UPPER LIMB IN CHRONIC STROKE

**DOI:** 10.2340/jrm.v58.44906

**Published:** 2026-04-21

**Authors:** Michiyuki KAWAKAMI, Daisuke ITO, Ken AZUMA, Takayuki KAMIMOTO, Arisa KAWABATA, Tetsuya TSUJI

**Affiliations:** Department of Rehabilitation Medicine, Keio University School of Medicine, Tokyo, Japan

**Keywords:** stroke, upper extremity, spasticity, botulinum toxin, rehabilitation

## Abstract

**Objective:**

This study aimed to investigate the association between botulinum toxin type A treatment and changes in spasticity and abnormal flexor synergies in patients with chronic stroke.

**Subjects:**

Twenty-eight patients with chronic stroke (mean age 56.5 years; mean time since onset 6.3 years) who received botulinum toxin type A injections into upper-arm flexor muscles (biceps brachii, brachialis, or brachioradialis) were enrolled.

**Methods:**

This was a retrospective, single-centre cohort study. Assessments were performed before and approximately 2 months after injection. Primary outcomes were changes in spasticity measured by the Modified Ashworth Scale and the abnormal flexor synergy index based on kinematic analysis. Secondary outcomes included changes in voluntary shoulder flexion angle and subgroup analyses according to baseline upper extremity motor severity and responder/non-responder status based on changes in the Modified Ashworth Scale scores of the elbow flexors.

**Results:**

Generalized estimating equation analyses showed significant changes in Modified Ashworth Scale elbow flexor scores and the abnormal flexor synergy index after injection. In severity-stratified analyses, significant Time × Severity interactions were observed for Modified Ashworth Scale scores of the elbow and wrist flexors, whereas no significant interaction was found for the abnormal flexor synergy index. In responder/non-responder analyses, a significant Time × Responder-status interaction was observed for the abnormal flexor synergy index. Non-responders showed no significant changes in Modified Ashworth Scale scores or the abnormal flexor synergy index and showed reduced maximum voluntary shoulder flexion angle.

**Conclusion:**

This study suggests that botulinum toxin type A treatment was associated with changes in abnormal flexor synergies, in addition to reducing muscle tone. These findings highlight the value of incorporating kinematic assessments such as the abnormal flexor synergy index into the clinical evaluation of botulinum toxin treatment.

## LAY ABSTRACT

Botulinum toxin injections are widely used to reduce muscle stiffness in people who have had a stroke. However, stroke can also cause abnormal movement patterns that make it difficult to control the arm during daily activities. It is not well known whether this treatment can improve these movement problems. In this study, we examined changes in arm movement before and after botulinum toxin injections in people with long-term stroke. We found that the treatment was associated not only with reduced muscle stiffness but also with changes in abnormal movement patterns. These findings suggest that botulinum toxin injections may help improve arm movement, and not just reduce muscle stiffness. This may help clinicians better evaluate treatment effects and plan rehabilitation.

Post-stroke spasticity is now understood as more than a simple increase in muscle tone. Recent work has redefined spasticity as a velocity- and muscle length-dependent increase in resistance to externally imposed stretch, resulting from hyperexcitability of descending brainstem pathways and exaggerated stretch reflexes (1). The overall prevalence of post-stroke spasticity has been reported to be 39.5% in patients after a first-ever stroke with motor paresis (2). In the chronic phase, spasticity is present in up to 97% of patients with moderate and severe motor impairments (3). Post-stroke spasticity is associated with reduced independence in activities of daily living, impaired health-related quality of life, and increased caregiver burden due to poorer physical and emotional health (4). Stroke survivors with spasticity also incur approximately fourfold higher healthcare costs in the first post-stroke year than those without spasticity (5). Thus, post-stroke spasticity represents a highly prevalent and socioeconomically burdensome complication requiring effective management.

Spasticity coexists with maladaptive motor impairments such as abnormal synergies, inappropriate co-activation, and inter-joint coupling (6). These abnormalities arise from disinhibition of the reticulospinal system following corticospinal tract injury, leading to increased excitatory descending inputs from the contralesional premotor and supplementary motor areas (6). Post-stroke spasticity is more prevalent in the upper limb than in the lower limb (7). Maladaptive movement patterns in the upper limb typically appear as abnormal flexor synergies, such as involuntary elbow flexion during shoulder flexion, as well as impaired force control and stereotyped multi-joint activation patterns (1, 6). As a result, upper limb spasticity is considered to interfere with the execution of normal motor behaviours across the acute-to-chronic phases and is associated with abnormal postures and joint contractures (1). Thus, post-stroke upper limb spasticity should be regarded as a complex neurophysiological syndrome that disrupts motor coordination and execution, rather than as an isolated increase in tone.

A variety of interventions are available for managing post-stroke spasticity, including physical therapy, oral antispasmodic medications, intrathecal baclofen, phenol neurolysis, and surgical procedures (1). For focal involvement of the upper limb, botulinum toxin type A (BTX-A) has become the treatment of choice due to its favourable safety profile and strong efficacy in reducing muscle overactivity (1). A systematic review and meta-analysis demonstrated that BTX-A significantly reduced muscle tone in the affected upper limb in patients after stroke or traumatic brain injury (8). A large, randomized, controlled trial, the BoTULS trial, also confirmed significant reductions in upper limb spasticity and pain with BTX-A; however, no significant differences were found in upper limb function (9). A recent trial similarly reported no improvement in upper limb activity with BTX-A monotherapy in chronic stroke (10). Thus, BTX-A is effective in alleviating upper limb spasticity, but because it primarily targets peripheral muscle overactivity rather than central neural mechanisms of recovery, its association with voluntary motor performance remains inconclusive. The inconsistent effects of BTX-A on upper-limb motor function may partly reflect differences in outcome measures across studies. While commonly used clinical assessments such as the Action Research Arm Test and the Box and Block Test have often failed to demonstrate significant functional improvement after BTX-A treatment (9, 10), improvements have been reported using instrumented assessments, including electromyographic indices of muscle activity (11–13) and kinematic parameters of upper-limb movement (14, 15). This discrepancy may be partly related to stroke-specific abnormal synergies that are not adequately captured by conventional functional scales. After stroke, pathological muscle co-activation and altered reciprocal inhibition arise due to central lesions impairing the corticospinal tracts (16). Consequently, even during voluntary single-joint movements, excessive and unintended motion frequently occurs in adjacent joints (17, 18). In the upper limb, the most common abnormal flexor synergy is involuntary elbow flexion during shoulder flexion (19,20), which represents a major contributor to impaired reaching ability after stroke (21, 22). This abnormal flexor synergy has been shown to be closely associated with upper-limb motor impairment but is not adequately reflected by conventional functional outcome measures, underscoring its importance when considering BTX treatment strategies (23). However, despite its potential clinical relevance, the relationship between botulinum toxin treatment and abnormal flexor synergy has not been systematically investigated. Therefore, this study aimed to investigate whether BTX-A treatment is associated with changes in abnormal flexor synergies in patients with chronic stroke. Given the observational pre–post design, potential confounding factors such as concomitant rehabilitation intensity, spontaneous recovery, and regression to the mean cannot be completely excluded.

## METHODS

### Study design and participants

**Fig. 1:**
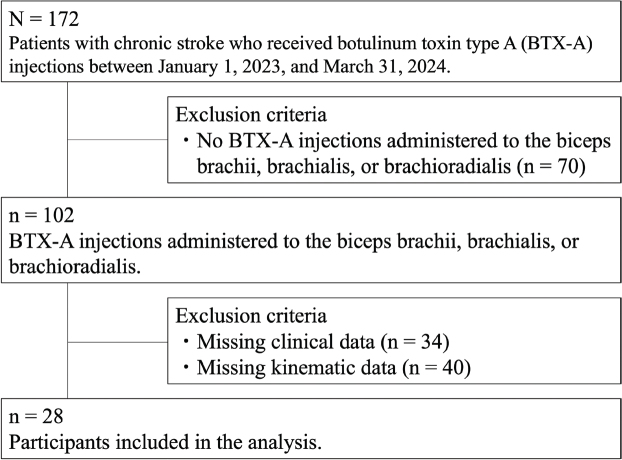
Flow diagram of study participants.

This retrospective, single-centre, uncontrolled cohort study was conducted in accordance with the STROBE checklist and used a convenience sample. Informed consent was obtained using an opt-out procedure. A total of 172 patients with chronic stroke who received BTX-A injections at Keio University Hospital between 1 January 2023, and 31 March 2024, were identified from the medical records. Patients who did not receive BTX-A injections in the biceps brachii, brachialis, or brachioradialis were excluded (*n* = 70). In addition, patients with missing clinical data (*n* = 34) or missing kinematic assessment data (*n* = 40) were excluded. Finally, 28 patients were included in the analysis (Fig. 1). Spasticity and kinematic assessments were performed both at baseline and approximately two months after the injection. The study was conducted in accordance with the Declaration of Helsinki and was approved by the Ethics Committee of Keio University (approval number: 20231066).

### Botulinum injection technique

Botulinum toxin injections were performed by a single physiatrist with more than 20 years of clinical experience and board-certified training in rehabilitation medicine. All injections were administered using onabotulinumtoxinA (ona-BoNT-A). Muscle localization was performed using electrical stimulation guidance alone; ultrasound and electromyographic guidance were not used. Electrical stimulation guidance has been reported to provide localization accuracy comparable to that of ultrasound or electromyographic guidance in limb spasticity management (24). Detailed procedural parameters such as the dilution ratio, total injected volume, and the number of injection sites per muscle were not consistently documented in the medical records.

### Data collection

Medical records were reviewed to collect demographic and clinical information, including sex, age, time since stroke onset, stroke type, side of hemiparesis, BTX-A dosage, history of prior BTX-A treatment, use of oral antispastic medications, and upper extremity motor function measured by the Fugl-Meyer Assessment of the Upper Extremity (FMA-UE) (25). The FMA-UE consists of 30 motor function items and 3 reflex items, each scored on a 3-point ordinal scale (0 = cannot perform the movement, 1 = performs partially, 2 = performs fully), with a total possible score of 0–66. Higher scores indicate better motor function. The FMA-UE is divided into 4 subsections: A, shoulder/elbow/forearm (0–36); B, wrist (0–10); C, hand (0–14); and D, coordination/speed (0–6).

### Outcome measures

The primary outcomes of this study were changes in elbow flexor spasticity and abnormal flexor synergy following BTX-A injection. Secondary outcomes included changes in the maximum voluntary shoulder flexion angle, spasticity of the wrist and finger flexors, and subgroup analyses based on upper-extremity motor severity and clinical response.

### Spasticity assessment

Upper extremity spasticity was assessed using the Modified Ashworth Scale (MAS) (26), which evaluates muscle tone in the elbow, wrist, and finger flexors (metacarpophalangeal [MP] and proximal interphalangeal [PIP] joints). The MAS is a 6-point ordinal scale (0, 1, 1+, 2, 3, and 4), with higher scores indicating more severe spasticity. All MAS evaluations were performed by physicians in the Department of Rehabilitation Medicine.

### Kinematic assessment

Kinematic data were obtained using a markerless motion capture system (Azure Kinect DK; Microsoft Corp, Redmond, WA, USA) during voluntary shoulder flexion. The reliability of this system for assessing upper limb movement in patients with stroke has been established previously, including shoulder and elbow joint kinematics under standardized measurement conditions (27). Previous validation studies comparing Azure Kinect with optical motion capture systems have reported small but systematic joint-angle differences (28). In the present study, measurements were performed under standardized conditions with a fixed camera distance and orientation. The Kinect recorded three-dimensional joint coordinates at a sampling frequency of 30 Hz. Participants sat approximately 2.5 m from the Kinect sensor, which was positioned at a height of 1 m, and performed maximal shoulder flexion twice while keeping the elbow fully extended. Repeated movement trials were used to derive the kinematic indices, which helped reduce the influence of random measurement variability.

As a preprocessing step, three-dimensional coordinate data for the shoulder (Sx, Sy, Sz), elbow (Ex, Ey, Ez), and hand (Hx, Hy, Hz) were acquired. These data were extracted using dedicated software (ICpro-K2; Hu-tech Co Ltd, Tokyo, Japan). Spline interpolation was applied to address missing data points, and the data were smoothed using a second-order Butterworth filter with a cut-off frequency of 5 Hz and exported for further analysis. Data were processed on a dedicated computer to calculate the maximum voluntary shoulder flexion angle and the abnormal flexor synergy index (29). The mean values of the 2 trials were used for all kinematic analyses to reduce random variability. Participants were instructed to keep the elbow as extended as possible during shoulder flexion in order to minimize voluntary elbow flexion. Under this instruction, any observed elbow flexion was interpreted as involuntary movement attributable to abnormal flexor synergy. The abnormal flexor synergy index quantifies the degree of involuntary elbow flexion during voluntary shoulder flexion. This parameter was derived from the three-dimensional coordinates of the shoulder, elbow, and hand as previously described (29). It reaches a maximum value of 100% when the elbow remains fully extended and decreases as involuntary elbow flexion increases during shoulder flexion. Lower values indicate a greater degree of abnormal synergy. This calculation method was adapted from a previously published protocol and has demonstrated excellent validity and responsiveness (29).

### Statistical analyses

Statistical analyses were performed using generalized estimating equations (GEE) with robust standard errors to evaluate pre- to post-BTX-A changes, accounting for within-participant correlations. Time (pre vs post) was included as a within-subject factor. For subgroup analyses, a Time × Group interaction term was added to examine whether changes over time differed by subgroup. For severity-based analyses, participants were stratified according to FMA-UE total scores: severe (< 20) and moderate (20–46) (30). For the response-based analyses, participants were classified into 2 groups according to changes in the MAS score of the elbow flexors after BTX-A injection: (1) an improved group (≥ 1-grade reduction) and (2) an unchanged group (no change). Estimated marginal means with 95% confidence intervals (95% CI) are reported for each time point and subgroup, and β represents the estimated mean change (post–pre). To control for multiplicity, *p*-values were adjusted using the Bonferroni method (within each table). Statistical analyses were conducted using IBM SPSS Statistics (version 30.0; IBM Corp, Armonk, NY, USA), with significance set at *p* ≤ 0.05.

## RESULTS

### Participants’ characteristics

The general characteristics of the participants are summarized in Table I. The mean age of the 28 participants was 56.5 (standard deviation (SD) = 9.4) years, and 22 were men. The median time since stroke onset was 6.3 years (interquartile range: 3.4–9.4). Stroke type was haemorrhagic in 23 participants and ischaemic in 5. Right and left hemiparesis were equally distributed (*n* = 14 each). The mean total FMA-UE score was 22.5 (SD = 8.1), with subsection scores of 18.4 (SD = 5.5) for A (shoulder/elbow/forearm), 1.5 (SD = 1.9) for B (wrist), 2.6 (SD = 2.5) for C (hand), and 0.0 (SD = 0.0) for D (coordination/speed).

**Table I:** General characteristics of the participants

Characteristics	Overall
Number	28
Sex (men/women)^a^	22/6
Age (years)^b^	56.5 (9.4)
Duration from stroke onset (years)^c^	6.3 (3.4–9.4)
Stroke type (haemorrhage/infarction)^a^	23/5
Paralysis side (right/left)^a^	14/14
FMA-UE, total score (0–66)^b^	22.5 (8.1)
A score (0–36)^b^	18.4 (5.5)
B score (0–10)^b^	1.5 (1.9)
C score (0–14)^b^	2.6 (2.5)
D score (0–6)^b^	0.0 (0.0)
Duration from pre- to post- BTX-A (days)^c^	46 (39–51)
BTX-A Biceps brachii (unit)^c^	50 (30–60)
BTX-A Brachialis (unit)^c^	45 (32.5–57.5)
BTX-A Brachioradialis (unit)^c^	45 (35–57.5)
BTX-A arm total (Biceps brachii + Brachialis + Brachioradialis) (unit)^c^	80 (47.5–120)
Prior BTX-A treatment history (naive/previously)^a^	0/28
Oral antispastic medication use, *n* (%)	4 (14.3)

a Number,

b mean (standard deviation),

c median (interquartile range).

BTX-A: botulinum toxin type A; FMA-UE: Fugl-Meyer assessment of the upper extremity.

### Changes in spasticity and kinematic outcomes following BTX-A injection

**Fig. 2:**
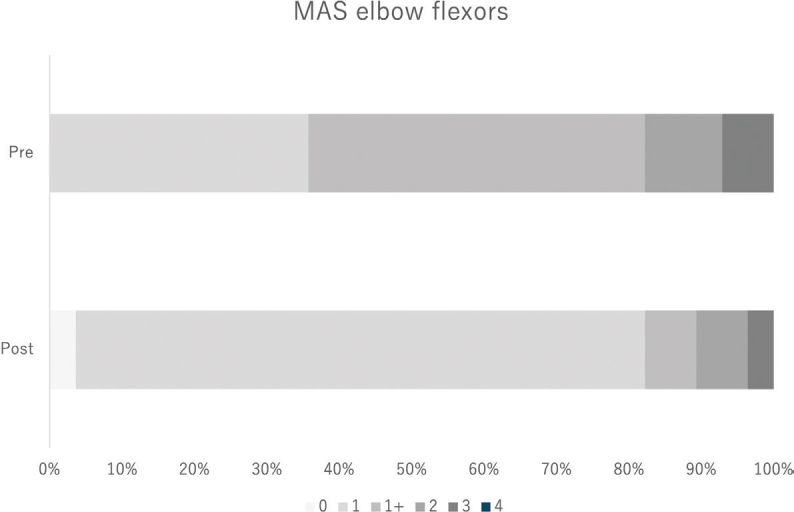
Shift in MAS elbow flexor categories before and after BTX-A injection.

**Fig. 3:**
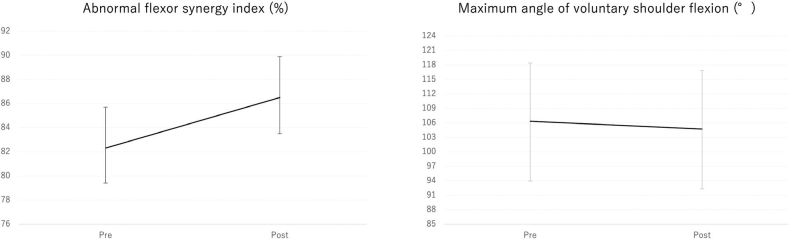
Changes in abnormal flexor synergy index and maximum voluntary shoulder flexion angle before and after BTX-A injection.

Table II presents the pre- to post-BTX-A injection changes in spasticity and kinematic outcomes. Significant reductions were observed in the MAS elbow flexor scores (β = –0.34, 95% CI: –0.47 to –0.21, adjusted *p* < 0.001) (Fig. 2) and MAS finger PIP flexor scores (β = –0.25, 95% CI: –0.39 to –0.11, adjusted *p* < 0.001). Although the MAS wrist flexor and finger MP flexor scores showed negative β coefficients, these changes did not remain significant after adjustment for multiple comparisons. Regarding kinematic outcomes, a significant change was observed in the abnormal flexor synergy index, which increased from 82.3% (95% CI: 78.9 to 85.7) to 86.5% (95% CI: 83.5 to 89.4) (β = 4.16, 95% CI: 2.16 to 6.17, adjusted *p* < 0.001), indicating reduced abnormal synergy during shoulder flexion (Fig. 3). In contrast, no significant change was observed in the maximum voluntary shoulder flexion angle (β = –1.58, 95% CI: –6.00 to 2.85, adjusted *p* = 1.000) (Fig. 3).

**Table II:** Comparison of MAS and kinematic parameters pre- and post- BTX-A injection

Outcome measures	Pre- BTX-A	Post-BTX-A	β (95% CI)	Wald χ²	Adjusted *p*-value
MAS, elbow flexors (0/1/1+/2/3/4)^a^	0/10/13/3/2/0	1/22/2/2/1/0	–0.34 (–0.47 to –0.21)	25.59	< 0.001
MAS, wrist flexors (0/1/1+/2/3/4)^a^	2/5/14/3/4/0	2/6/15/5/0/0	–0.20 (–0.36 to –0.03)	5.34	0.126
MAS, finger MP flexors (0/1/1+/2/3/4)^a^	8/7/10/3/0/0	10/11/5/2/0/0	–0.20 (–0.38 to –0.02)	4.54	0.198
MAS, finger PIP flexors (0/1/1+/2/3/4)^a^	1/4/17/4/2/0	1/11/13/3/0/0	–0.25 (–0.39 to –0.11)	13.01	< 0.001
Maximum angle of voluntary shoulder flexion (°)^b^	106.3 (94.2–118.4)	104.7 (92.3–117.1)	–1.58 (–6.00–2.85)	0.49	1.000
Abnormal flexor synergy index (%)^b^	82.3 (78.9–85.7)	86.5 (83.5–89.4)	4.16 (2.16–6.17)	16.52	< 0.001

a Number,

b estimated marginal mean (95% CI).

Generalized estimating equations with robust standard errors were used to evaluate pre–post changes.

β represents the estimated mean change (Post–Pre) derived from generalized estimating equations.

MAS scores were analysed as continuous variables in the GEE model.

*P*-values were adjusted for multiple comparisons using the Bonferroni method.

BTX-A: botulinum toxin type A; CI: confidence interval; MAS: Modified Ashworth Scale; MP: metacarpophalangeal; PIP: proximal interphalangeal.

### Subgroup analysis by severity of upper-extremity paralysis

Table III lists the characteristics of participants classified by severity. Twelve participants were assigned to the severe group (FMA-UE < 20) and 16 to the moderate group (FMA-UE 20–46). No significant group differences were found in basic characteristics other than FMA-UE.

**Table III:** Participants’ characteristics by severity of upper extremity paralysis

Characteristics	Severe group	Moderate group	*p*-value
Number	12	16	–
Sex, men/women, *n*	9/3	13/3	0.802
Age, years, mean (SD)	57.0 (9.2)	56.2 (9.6)	0.537
Duration from stroke onset, years, median (IQR)	6.8 (3.4–9.5)	5.9 (3.5–8.5)	0.631
Stroke type, haemorrhage/infarction, *n*	10/2	13/3	0.945
Paralysis side, right/left, *n*	6/6	8/8	1.000
FMA-UE, total score, 0–66, mean (SD)	14.7 (1.4)	28.3 (5.7)	< 0.001
A score (0–36)	12.8 (2.3)	22.6 (2.7)	< 0.001
B score (0–10)	0.6 (1.0)	2.1 (2.1)	0.053
C score (0–14)	1.3 (0.7)	3.6 (2.9)	0.006
D score (0–6)	0.0 (0.0)	0.0 (0.0)	1.000
Duration from pre- to post- BTX-A, days, median (IQR)	43 (41–46)	50 (38–53)	0.260
BTX-A Biceps brachii, unit, median (IQR)	50 (30–60)	40 (24–67.5)	0.302
BTX-A Brachialis, unit, median (IQR)	50 (40–80)	40 (35–50)	0.909
BTX-A Brachioradialis, unit, median (IQR)	65 (57.5–72.5)	30 (25–35)	0.802
BTX-A arm total (Biceps brachii + Brachialis + Brachioradialis), unit, median (IQR)	85 (45–125)	70 (47.5–97.5)	0.478
Prior BTX-A treatment history, naive/previously, *n*	0/12	0/16	1.000
Oral antispastic medication use, *n* (%)	3 (25.0)	1 (6.3)	0.423

Mann–Whitney *U* test used for between-group comparisons.

SD: standard deviation; IQR: interquartile range; BTX-A: botulinum toxin type A; FMA-UE: Fugl-Meyer assessment of the upper extremity.

Table IV presents the results of GEE models examining time effects and Time × Severity interactions. A significant Time × Severity interaction was observed for MAS elbow flexors (β = –0.35, 95% CI: –0.60 to –0.11, adjusted *p* = 0.030) and MAS wrist flexors (β = –0.46, 95% CI: –0.79 to –0.13, adjusted *p* = 0.036), indicating greater reductions in spasticity in the severe group compared with the moderate group. No significant interaction effects were observed for MAS finger MP flexors, MAS finger PIP flexors, maximum voluntary shoulder flexion angle, or the abnormal flexor synergy index after adjustment for multiple comparisons.

**Table IV:** Pre- and post-BTX-A values and Time × Severity interactions for each outcome measure

Outcome measures	Severe group (*n* = 12)		Moderate group (*n* = 16)		Interaction β (95% CI)	Wald χ²	Adjusted *p*-value
	Pre	Post	Pre	Post			
MAS, elbow flexors (0/1/1+/2/3/4)^a^	0/2/5/3/2/0	1/8/0/2/1/0	0/8/8/0/0/0	0/14/2/0/0/0	–0.35 (–0.60 to –0.11)	8.01	0.030
MAS, wrist flexors (0/1/1+/2/3/4)^a^	0/1/6/1/4/0	0/2/7/3/0/0	2/4/8/2/0/0	2/4/8/2/0/0	–0.46 (–0.79 to –0.13)	7.54	0.036
MAS, finger MP flexors (0/1/1+/2/3/4)^a^	3/1/5/3/0/0	4/4/2/2/0/0	5/6/5/0/0/0	6/7/3/0/0/0	–0.17 (–0.52–0.18)	0.87	1.000
MAS, finger PIP flexors (0/1/1+/2/3/4)^a^	1/0/7/2/2/0	1/4/5/2/0/0	0/4/10/2/0/0	0/7/8/1/0/0	–0.29 (–0.57 to –0.02)	4.32	0.228
Maximum angle of voluntary shoulder flexion (°)^b^	72.7 (62.5–82.8)	73.9 (62.3–85.5)	131.5 (125.5–137.6)	127.8 (118.1–137.6)	4.90 (–3.27–13.06)	1.38	1.000
Abnormal flexor synergy index (%)^b^	75.4 (71.6–79.1)	80.7 (76.3–85.1)	87.5 (84.0–91.0)	90.8 (88.6–93.0)	2.05 (–2.23–6.33)	0.88	1.000

a Number,

b estimated marginal mean (95% CI).

Generalized estimating equations with robust standard errors were used to evaluate changes over time and Time × Severity interactions.

β represents the estimated mean change (Post–Pre) derived from the generalized estimating equations.

Estimated marginal means (95% CI) are presented for each group and time point.

MAS scores were analysed as continuous variables in the GEE model.

*P*-values for interaction terms were adjusted for multiple comparisons using the Bonferroni method.

BTX-A: botulinum toxin type A; MAS: Modified Ashworth Scale; MP: metacarpophalangeal; PIP: proximal interphalangeal.

### Comparison of responders and non-responders

Participants were categorized into 2 subgroups based on changes in the MAS elbow flexors: the MAS-improved group (*n* = 15) and the MAS-unchanged group (*n* = 13). As indicated in Table V, no significant differences were observed between the 2 groups.

**Table V:** Participants’ characteristics by clinical response subgroups

Characteristics	Responder: MAS elbow flexors improved group	Non-responder: MAS elbow flexors unchanged group	*p*-value
Number	15	13	–
Sex, men/women, *n*	11/4	11/2	0.618
Age, years, mean (SD)	58.7 (9.0)	54.0 (9.3)	0.525
Duration from stroke onset, years, median (IQR)	6.2 (3.5–8.1)	7.1 (3.4–10.5)	0.618
Stroke type, haemorrhage/infarction, *n*	13/2	10/3	0.683
Paralysis side, right/left, *n*	8/7	6/7	0.751
FMA-UE, total score, 0–66 mean (SD)	21.1 (8.5)	24.1 (7.2)	0.316
A score (0–36)	16.7 (5.6)	20.2 (4.8)	0.156
B score (0–10)	1.6 (2.2)	1.3 (1.4)	0.856
C score (0–14)	2.7 (2.7)	2.5 (2.3)	1.000
D score (0–6)	0.0 (0.0)	0.0 (0.0)	1.000
Duration from pre- to post- BTX-A, days, median (IQR)	46 (41–49)	46 (39–56)	0.555
BTX-A Biceps brachii, unit, median (IQR)	50 (32.5–60)	30 (30–50)	0.065
BTX-A Brachialis, unit, median (IQR)	40 (30–50)	50 (40–75)	0.683
BTX-A Brachioradialis, unit, median (IQR)	50 (35–65)	45 (42.5–47.5)	0.928
BTX-A arm total (Biceps brachii + Brachialis + Brachioradialis), unit, median (IQR)	80 (50–110)	80 (30–120)	0.440
Prior BTX-A treatment history, naïve/previously, *n*	0/15	0/13	1.000
Oral antispastic medication use, *n* (%)	4 (26.7)	0 (0.0)	0.235

Mann–Whitney *U* test used for between-group comparisons.

SD: standard deviation; IQR: interquartile range; BTX-A: botulinum toxin type A; FMA-UE: Fugl-Meyer assessment of the upper extremity.

Table VI summarizes the results of the GEE models examining Time × Responder-status interactions. A significant interaction effect was observed for MAS elbow flexors (β = −0.63, 95% CI: −0.75 to −0.52, adjusted p < 0.001), confirming greater reductions in spasticity in the MAS-improved group compared with the unchanged group. A significant interaction was also found for the abnormal flexor synergy index (β = 4.99, 95% CI: 1.41 to 8.57, adjusted *p* = 0.036), indicating a larger change in the abnormal flexor synergy index in the MAS-improved group. Although the maximum voluntary shoulder flexion angle showed a nominal interaction effect (β = 10.87, 95% CI: 2.65 to 19.10, *p* = 0.010), this did not remain significant after adjustment for multiple comparisons (adjusted *p* = 0.060). No significant interaction effects were observed for MAS wrist, finger MP, or finger PIP flexors after correction.

**Table VI:** Pre- and post-BTX-A values and Time × Responder-status interactions for each outcome measure

Outcome measures	Responder: MAS elbow flexors improved group (*n* = 15)		Non-responder: MAS elbow flexors unchanged group (*n* = 13)		Interaction β (95% CI)	Wald χ²	Adjusted *p*-value
	Pre	Post	Pre	Post			
MAS, elbow flexors (0/1/1+/2/3/4)^a^	0/1/11/2/1/0	1/13/0/1/0/0	0/9/2/1/1/0	0/9/2/1/1/0	–0.63 (–0.75 to –0.52)	123.07	< 0.001
MAS, wrist flexors (0/1/1+/2/3/4)^a^	1/2/8/2/2/0	1/3/9/2/0/0	1/3/6/1/2/0	1/3/6/3/0/0	–0.08 (–0.41–0.25)	0.22	1.000
MAS, finger MP flexors (0/1/1+/2/3/4)^a^	6/1/6/2/0/0	7/5/1/2/0/0	2/6/4/1/0/0	3/6/4/0/0/0	–0.08 (–0.43–0.27)	0.20	1.000
MAS, finger PIP flexors (0/1/1+/2/3/4)^a^	1/1/10/2/1/0	1/5/8/1/0/0	0/3/7/2/1/0	0/6/5/2/0/0	–0.04 (–0.31–0.24)	0.07	1.000
Maximum angle of voluntary shoulder flexion (°)^b^	97.0 (80.1–114.0)	100.5 (82.6–118.3)	117.0 (101.7–132.4)	109.6 (93.1–126.2)	10.87 (2.65–19.10)	6.71	0.060
Abnormal flexor synergy index (%)^b^	80.1 (75.7–84.4)	86.5 (82.9–90.1)	84.9 (79.9–89.8)	86.4 (81.7–91.1)	4.99 (1.41–8.57)	7.45	0.036

a Number,

b estimated marginal mean (95% CI).

Generalized estimating equations with robust standard errors were used to evaluate changes over time and Time × Responder-status interactions.

β represents the estimated mean change (Post–Pre) derived from the generalized estimating equations.

Estimated marginal means (95% CI) are presented for each group and time point.

MAS scores were analysed as continuous variables in the GEE model.

*P*-values for interaction terms were adjusted for multiple comparisons using the Bonferroni method.

BTX-A: botulinum toxin type A; MAS: Modified Ashworth Scale; MP: metacarpophalangeal; PIP: proximal interphalangeal.

## DISCUSSION

This study found that BTX-A treatment was associated with changes in abnormal flexor synergies in patients with post-stroke upper limb spasticity. These findings suggest that BTX-A may have potential impacts beyond reducing spasticity and may also be associated with changes in movement patterns.

BTX-A was associated with significant changes not only in the MAS scores of the elbow, wrist, and finger flexors, but also in the abnormal flexor synergy index. The improvement in MAS scores was consistent with numerous previous studies (8–10), reaffirming that BTX-A reduces spasticity. Importantly, this study also identified significant changes in the abnormal flexor synergy index, suggesting a reduction in abnormal flexor synergies. The improvement in motor function with BTX-A remains controversial, and some previous studies have reported that BTX-A does not contribute to improvements in upper limb function (9, 10). Previous instrumented assessments, including electromyographic indices of muscle activity and kinematic parameters of upper-limb movements, have reported BTX-A-related changes in movement characteristics (11–15). However, these studies did not specifically quantify the relationship between such changes and stroke-specific abnormal flexor synergies. In contrast, the abnormal flexor synergy index is a quantitative measure used to assess involuntary elbow flexion during shoulder flexion (29). As a kinematic surrogate marker, it may reflect a specific aspect of spasticity-related motor impairment that is not fully captured by conventional clinical scales. Against this background, the present study quantitatively describes the changes in abnormal flexor synergies following BTX-A treatment, providing additional insight into the relationship between spasticity reduction and movement patterns.

Subgroup analysis based on motor impairment severity showed significant changes in both MAS elbow flexors and the abnormal flexor synergy index across the severe and moderate groups. Previous studies have indicated that BTX-A reduces spasticity effectively in patients with moderate to severe hemiparesis, with these effects potentially independent of residual motor function (31). The present findings are consistent with this notion. Regarding motor function, although a clear consensus has not been established, it has been suggested that improvements in motor function after BTX-A depend on residual motor capacity (32). In this context, the observation that changes in the abnormal flexor synergy index were detected in both severity groups should be interpreted cautiously, as these subgroup analyses were exploratory. However, the present study demonstrated that significant changes in the abnormal flexor synergy index were observed regardless of severity, with severe patients benefiting to a similar extent as patients with moderate impairment. This pattern may be consistent with previous studies suggesting that central lesions involving the corticospinal tract are associated with pathological co-activation and altered reciprocal inhibition after stroke (20), which may contribute to abnormal flexor synergies. Therefore, such synergies may not necessarily follow a linear relationship with voluntary motor control. These results indicate that BTX-A treatment was associated with changes in abnormal flexor synergies independently of stroke severity, although this interpretation requires confirmation in prospective studies.

Subgroup analysis based on improvements in MAS elbow flexors showed differences between responders and non-responders. In the responder group, significant changes were found in MAS elbow flexors, MAS finger PIP flexors, and the abnormal flexor synergy index. In contrast, the non-responder group showed no significant changes in MAS scores or the abnormal flexor synergy index and instead exhibited a significant reduction in maximum voluntary shoulder flexion angle. Previous studies have reported that, although BTX-A reduces spasticity, this does not necessarily translate into improvements in motor function (9, 10). The present study demonstrated that spasticity and abnormal flexor synergies can change in tandem, highlighting a potential interaction between these pathological features. However, because the responder definition in this study was based on changes in MAS elbow flexor scores, the findings of this subgroup analysis should be interpreted as descriptive and hypothesis-generating rather than confirmatory. Importantly, the present study also showed that some non-responders experienced a decline in voluntary motor function. Although BTX-A is generally considered safe, unwanted weakness in injected muscles has been reported (33), with 1 study reporting post-injection muscle weakness in up to 19% (34). Thus, despite the intended antispastic effects of BTX-A, the decreased voluntary motor function observed in some patients in the present study is not unexpected. In the present study, the target muscles included the biceps brachii, brachialis, or brachioradialis. Of the injected muscles, the biceps brachii also contributes to shoulder flexion, and injections into this muscle could have contributed to the noted decrease in voluntary shoulder flexion. In addition, measurement variability and regression to the mean may also partly explain the observed decrease in maximum voluntary shoulder flexion angle in this subgroup. However, few studies have objectively quantified such changes, and reporting these data may provide additional information for clinical decision-making.

In addition, no significant differences were found between responders and non-responders in baseline characteristics such as age, sex, duration from stroke onset, and FMA-UE scores, making it difficult to predict responsiveness from these variables. Few studies have systematically examined predictors of responsiveness to BTX-A based on individual patient factors, and the underlying mechanisms remain unclear. One proposed explanation for an apparent decrease in BTX-A efficacy over time is the development of neutralizing antibodies. However, the reported prevalence of neutralizing antibodies varies widely, ranging from 0.9% to 13.9% (35, 36). Moreover, the presence of such antibodies does not always predict treatment non-response, since at least some patients with neutralizing antibodies retain normal sensitivity, whereas many patients deemed clinically non-responsive do not have detectable neutralizing antibodies (37). Thus, a clear consensus regarding the frequency and clinical impact of neutralizing antibodies has not been established. Instead, non-immunological factors are considered more clinically relevant. After a stroke, progressive pathological changes such as muscle fibrosis and fatty infiltration are known to occur over time (38), and muscles with high baseline echo intensity have been reported to show worse outcomes following BTX-A treatment (39). These findings suggest that structural changes within the muscle may play a crucial role in determining responsiveness to BTX-A. Future studies should accumulate data incorporating these evaluation parameters to clarify the clinical characteristics of responders. However, the therapeutic targets of BTX-A extend beyond spasticity reduction and include pain, activity limitations, and quality-of-life impairments associated with spasticity (40, 41). Therefore, the non-responder group defined in the present study should not be regarded as a group for whom BTX-A is not indicated. Indeed, patient satisfaction with BTX-A treatment has been reported to be high (40, 42). Future investigations should also assess changes in spasticity-related symptoms and patient-reported satisfaction, together with clinical outcomes.

The findings of the present study provide new insights into the clinical application of BTX-A. This study is the first to quantitatively demonstrate that BTX-A treatment was associated with changes in abnormal flexor synergies, suggesting that the clinical impact of BTX-A may extend from reducing muscle tone to changes in movement patterns. The abnormal flexor synergy index can be obtained quickly and objectively using simple markerless motion capture and automated processing (29). Although markerless motion capture systems may exhibit joint-angle differences compared with laboratory-based optical motion capture systems, previous validation studies have reported that these differences are generally small but systematic (28). Because the present study focused on within-subject pre–post comparisons performed under identical standardized conditions, such systematic errors were unlikely to materially influence the direction of the observed changes. Therefore, the evaluation of BTX-A should incorporate kinematic assessments such as the abnormal flexor synergy index along with conventional spasticity measures.

### Limitations

This study has several limitations. The first is its retrospective, observational design, which precludes definitive conclusions regarding causality. Second, the sample size was relatively small (*n* = 28), and the study was conducted at a single centre, limiting the generalizability of the findings. In addition, the cohort consisted predominantly of patients in the chronic stage of stroke with a long median time since onset; therefore, the findings may not be generalizable to subacute or acute stroke populations. Third, information on concomitant rehabilitation intensity, orthotic use, joint contracture status, the interval since previous BTX-A injections, and detailed procedural parameters of botulinum toxin administration (e.g., dilution ratio, injected volume, and number of injection sites per muscle) was not consistently available in this retrospective outpatient cohort. Therefore, their potential influence on treatment outcomes could not be fully assessed. Fourth, within-study reliability indices, such as intra-rater reliability, standard error of measurement, or minimal detectable change, were not calculated because this study relied on routinely collected clinical data; therefore, the smallest detectable change for the abnormal flexor synergy index could not be determined. Accordingly, the clinical relevance of the observed change in the abnormal flexor synergy index and responder classification based on this index could not be established in the present study. Moreover, because the evaluation was performed only once, approximately 2 months after injection, the long-term durability of the effects remains unclear. Finally, the abnormal flexor synergy index used in this study focuses specifically on elbow flexion during shoulder flexion and therefore provides a limited interpretation of abnormal synergies. However, this study targeted a homogeneous cohort of patients who exhibited spasticity in elbow flexors and received BTX-A injections into proximal upper limb muscles, making the interpretation of results more straightforward. Furthermore, this study is the first to quantitatively demonstrate that BTX-A treatment was associated with changes in abnormal flexor synergies, thereby expanding its therapeutic significance from merely reducing muscle tone to potentially normalizing movement patterns. Future multicentre, prospective studies with larger cohorts are warranted to validate these findings and further identify the clinical characteristics of responders.

### Conclusion

This retrospective single-centre cohort study suggests that BTX-A treatment was associated with changes in the abnormal flexor synergy index derived from kinematic analysis, in addition to reducing muscle tone, in patients with post-stroke upper limb spasticity. These findings highlight the novel relationship between spasticity and synergies, underscoring the clinical value of incorporating kinematic measures such as the abnormal flexor synergy index into the evaluation of the therapeutic effects of BTX-A. However, given the retrospective design, potential confounding factors, and measurement-related constraints, these findings should be considered preliminary and require confirmation in prospective, controlled studies evaluating both kinematic and functional outcomes.
